# #WedontWantDistanceEducation: a thematic analysis of higher education students’ social media posts about online education during Covid-19 pandemic

**DOI:** 10.1007/s10758-022-09621-x

**Published:** 2022-08-30

**Authors:** Muhterem Dindar, Ismail Çelik, Hanni Muukkonen

**Affiliations:** 1 Faculty of Education and Culture, Tampere University, FI-33014 Tampere, Finland; 2 Faculty of Education, University of Oulu, Oulu, Finland

**Keywords:** Covid-19, Distance education, Online education, Digital inequality, Higher Education

## Abstract

The current study is based on thematic analysis of 21,722 tweets posted under the #wedontwantdistanceeducation hashtag within a month after the start of online distance education in Turkish universities due to Covid-19 pandemic. Our findings have revealed that Turkish higher education students have faced multiple challenges in accessing and benefiting from online education due to the swift transformation from face-to-face to online format. These challenges included universities’ poor technical infrastructure, pedagogical and assessment issues, digital inequality in accessing online education, and general negative attitude towards online education. Further, students have expressed issues about financial, health, and social consequences of online education during Covid-19 pandemic. With regards to such challenges and issues, higher education students have criticized government authorities for ignoring their views when making decisions about how online learning is organized during Covid-19 pandemic. Further, students have offered some alternative solutions (e.g. summer courses) to online education.

## Introduction

Widespread use of social media in the modern society has created new opportunities for establishing open, dynamic and real-time communication between different layers of the public sphere. Specifically, social media has moved the communication between citizens and governments to a new level. Nowadays, many citizens actively use social media platforms to establish connections with the government authorities on civic and political issues rather than engaging with them through printed petitions or forms (Panagiotopoulos et al., 2014). The collaborative characteristics of social media also help citizens to raise their voices on public issues collectively (Author). Considering the transformative impact of social media on the public discourse, government agencies have been enthusiastically exploring methods to establish meaningful relationships with the society in social media platforms (Chen et al., 2020).

In higher education contexts, a plethora of studies have explored the potential of social media in pedagogical practices, networking and community building (Eaton & Pasquini, 2020). However, research has shown that higher education students use social media for civic and political issues related to education as well. For example, social media was found to play a significant role in mobilization of Chilean higher education students on the streets against the cost and quality of education in the country (Scherman et al., 2015). Austrian students used social media platforms to organize street protests against educational policy changes in higher education (Maireder & Schwarzenegger, 2012). In Canada, social media was an essential component of student-led strikes against high tuition fees at the universities (Raynauld et al., 2016). Further, it has been found that social media constitutes a valuable space for students activists in raising awareness about issues at the university campuses such as sexual violence, and cultural diversity (Cabrera et al., 2017; Mwangi et al., 2018).

People’s engagement on social media is highly situated and emerge “*within a specific historical, social, political and economic context*” (Sloan & Quan-Haase, 2017). Thus, social media can provide valuable insights about public’s stance on societal issues, specifically in times of disaster or crisis. In the last few years, the world has been going through tough times due to COVID-19 pandemic. Millions of people, if not billions, have been living in isolation. Due to social distancing necessity, public schools and universities have been closed in many countries. Turkey is one them. All public and private universities in the country suspended on-campus teaching and switched to online education with the decree from Turkish Higher Education Council on 26th of March 2020. The same day, #wedontwantdistanceeducation (#uzaktanegitimistemiyoruz) hashtag in Twitter became a trending topic in Turkey. The hashtag has become a venue for Turkish higher education students to express their questions, concerns and tensions related to the uncertainties around their education in a crisis.

The pandemic has imposed new demands and stressors on students (Coman et al., 2020; Conrad et al., 2021). Consequently, students’ response to remote online education during the pandemic has become a growing concern. Drawing on this, the aim of this research is to understand higher education students’ views and concerns on remote online education during the Covid-19 pandemic. Based on this aim, the current study presents a thematic analysis of tweets posted under the #wedontwantdistanceeducation hashtag within one month after the suspension of face-to-face teaching at Turkish universities. The current study highlights multiple challenges faced by the students, teachers and institutions due to abrupt switching to online education. Further, the study underlines that social media platforms can provide valuable information to educational policy makers about the acceptance and effectiveness of the ongoing educational transformations in higher education. Our research contributes to the knowledge base on understanding higher education students’ needs during crisis conditions. Further, it contributes to the development of effective online education systems that are resilient to abrupt disruptions in the educational landscape. In the following, we first present the state-of-art literature on the use of online formats in higher education and its challenges. We then present our research goals and methodological approach in pursuing them. The paper continues with presenting our findings and discussing them in relation to the relevant literature. The paper ends with the concluding remarks.

## Literature review

The advent of internet in 1990s have transformed Distance Education and gave birth to online education (Palvia et al., 2018). Since then, online education has become increasingly popular among the higher education institutions across the globe. For example, in United States, the number of students enrolled in online education programs have been steadily increasing for more than a decade and reached to 6 million in 2016 (Seaman et al., 2018). In India, online education attracted over 1,5 million learners in 2016, and is expected to attract over 9 million learners in 2021 (KPMG & Google, 2017). In China, online education market has reached to 120 million users in 2016 ( Zheng 2017). In Turkey, the biggest open education university in the country (i.e. Anadolu University) currently has more than 2 million students enrolled in various degree programs (Anadolu, 2020). However, online education has penetrated higher education systems to a limited extent in some parts of the world (e.g. Africa) due to low internet connectivity and poor technical infrastructure (Mathew & Ebelelloanya, 2016). Nevertheless, it seems that the trend in profound adoption of online education at higher education institutions will continue worldwide (Palvia et al., 2018).

The quality and effectiveness of online education has been scrutinized to a great extend in the literature. Several meta-analyses have found that students in online education conditions perform better than their counterparts in face-to-face education conditions (Ebner & Gegenfurtner, 2019; Means et al., 2013; Pei & Wu, 2019). Some studies found no difference between online and face-to-face education in terms of learning outcomes (Bowen et al., 2014; Driscoll et al., 2012). Other recent studies have found low learning achievement in online learning compared to face-to-face learning (Francis et al., 2019). Further, higher drop-out rates were observed in online higher education programs than their face-to-face counterparts (Glazier, 2016). It is worth noting that a growing body of teachers has been mixing online and face-to-face instruction at higher education courses. This mixing is commonly referred to as blended, hybrid, flipped or inverted learning (Margulieux et al., 2016). A meta-analysis by Spanjers and colleagues (2015) have shown that mixing face-to-face education with online education is more effective than solely face-to-face education. Considering these findings, it can be concluded that merely switching to online education does not guarantee successful learning.

With regard to satisfaction, studies generally reported higher learner satisfaction in face-to-face education than online education (Ebner & Gegenfurtner, 2019; Owston & York, 2018). The literature presents several key factors contributing to increased learner satisfaction in online learning. These factors include effective instructional design (Li et al., 2017), teachers’ pedagogical and technical skills (Rienties et al., 2012), presenting quality instructional content (Naveh et al., 2010), giving a manageable workload to the students (Li et al., 2016), facilitating interaction between educator and student (De Paepe et al., 2018), facilitating interactions among the learners (Kurucay & Inan, 2017), providing feedback during learning (van Popta et al., 2017), and applying diverse assessment methods (Sun et al., 2008). In addition to the factors summarized here, online education has some innate challenges.

Overall, there has been a negative view among the learners towards online education. This is partly due to the legacy of public distance education programs that require minimal or no admission requirements (Latchem et al., 2006). Thus, the qualifications received through distance education programs (extensively online, nowadays) have been questioned in the society (Gaskell & Mills, 2014). Further, there have been concerns about quality control in distance education (Casey, 2008). Although significant advancements made in terms of quality assurance in online education (Vlachopoulos, 2016), face-to-face education is still somehow perceived superior to online education (Grossman & Johnson, 2016).

Online education is essentially based on utilization of digital technologies and internet connection for teaching and learning. Therefore, a capable technological infrastructure is crucial for successful online education in higher education. This infrastructure includes hardware systems, course delivery and learning management systems, data management and learner support systems (Dahlstrom et al., 2014; Saba & Shearer, 2017). A challenge here is that development and maintenance of technical infrastructure for online education can be costly depending on the number of students enrolled in the system. In addition, it has been found that preparing course content for technologically advanced online courses cost more money than face-to-face courses (Poulin & Straut, 2017). Considering this, many universities have been looking for cost-effective solutions such as open-source learning management systems and open educational resources (Winitzky-Stephens & Pickavance, 2017). Nevertheless, research have shown that not all universities have sufficient technological infrastructure and financial capacity in providing quality online education (Ali et al., 2018).

Online education has been found to serve mostly to highly educated individuals with high socio-economic status (Hansen & Reich, 2015). In many regions, students lack suitable digital technologies (e.g. computers) and internet connectivity in accessing online education (Hillier, 2018). Thus, it has been claimed that online education has been sharpening inequalities in the society rather than providing better learning for the disadvantaged (Fischer et al., 2020). Considering this, there has been a growing discourse among the policy makers about how to remedy digital divide in the online education arena across the world (Patru & Balaji, 2016). Yet, digital divide in accessing education remains a challenge even in most developed countries (Cruz-Jesus et al., 2016).

According to socio-cognitive view, interaction is a crucial process for successful learning. Basic interactional processes in learning has been defined as learner-learner, learner-teacher, and learner-content interaction (Moore, 1989). Online education is a computer-mediated communication process whereby interactions among students, teachers and the course content occurs through digital means. For maximum effectiveness, online education should stimulate interactions among students, teachers and the course content (Jaggars & Xu, 2016). However, online learning environments have been mostly criticized for deficient interaction, feeling of isolation, loneliness and lack of participation have been a major reason for quitting online education (Tsiotakis & Jimoyiannis, 2016). Considering this, there has been a growing emphasis on enhancing interactivity in online learning settings (Diep et al., 2017;, Muukkonen et al., 2020).

To summarize, it is possible to provide high quality learning experiences in online platforms. However, this is not easy to achieve and online education providers face a variety of challenges in decreasing costs, increasing access and supporting learning. Despite these challenges, the interest in online education has been on steady increase in higher education contexts. Currently, higher education institutions are experiencing a massive shift towards online education due to the Covid-19 pandemic. Many universities across the world have halted their face-to-face education and switched to online education in a very short period. The impact of this massive and abrupt switch in higher education is yet to be explored from multiple perspectives. Drawing on this, the current study aims to bring out higher education students’ views and concerns about online education in time of Covid-19. Specifically, the current study focuses on Turkish higher education students’ Twitter posts related to online education during the Covid-19 pandemic.

## Methodology

On 10th of March 2020, Turkish Ministry of Health announced the first Covid-19 case in Turkey (Anadolu Ajansi, 2020a). On 12th of March 2020, Turkish Higher Education Council paused education in all universities in the country for three weeks starting on 16th of March (Anadolu Ajansi, 2020c). After the announcement, many university students rushed to bus stations to spend the “three-week holiday” in their hometowns (Haberturk, 2020). On 18th of March, Turkish Higher Education Council announced that face-to-face education in all public and private universities were suspended for the spring 2020 (Anadolu Ajansi, 2020b). Further, the council announced that universities would switch to distance education and continue their education in the online format starting 26th of March. Following the announcement, #wedontwantdistanceeducation (i.e. #uzaktanegitimistemiyoruz, in Turkish) hashtag appeared in Twitter on 18th of March, 2020. On 26th of March, the hashtag became one of the most discussed topics (i.e. trending topic) in Turkish Twitter.

With the assistance from Vicinitas online Twitter history tracking platform (https://www.vicinitas.io), we downloaded all the tweets (excluding retweets) posted under #wedontwantdistanceeducation hashtag between 18th of March 2020 and 23rd of April 2020. The dataset included 21,722 tweets posted by 15,858 unique Twitter accounts.

As a first step, a screening was conducted on the whole dataset to identify tweets (i.e. spams, commercials, news) that are not related to the topic of interest in this study (Author). Based on this screening, 1396 tweets were removed from the dataset. It was also observed that 4154 tweets were blank endorsement of #wedontwantdistanceeducation hashtag with no content. These blank endorsement tweets were excluded from the dataset as well. In the end, 16,172 tweets remained for further analyses.

A thematic analysis was conducted to characterize #wedontwantdistanceeducation tweets under specific themes. Thematic analysis is a common method for detecting and reporting patterns within data (Braun & Clarke, 2006). In the current study, the thematic analysis was conducted at the explicit level (Boyatzis, 1998). That is, tweets were interpreted within the explicit meanings of their content. We did not look for meanings that is beyond what people have posted (Author). Considering the extraordinary impact of the Covid-19 pandemic on education, we embraced a data-driven approach in developing the themes rather than analyzing tweets from a specific theoretical perspective. A six-step approach was followed in the analysis: (1) familiarize with the data; (2) generate initial codes; (3) look for themes; (4) refine themes; (5) name themes; (6) report themes (Braun & Clarke, 2003). Considering the big sample size, we started the analysis with randomly chosen 1000 tweets from the whole dataset. The first and the second author mutually coded this sub-sample and decided on the initial themes. Then, the second author coded the whole dataset based on the initial themes that emerged from the sub-sample. When coding the whole sample, themes were refined and updated whenever necessary. It was observed that a single tweet might reflect views that are related to more than one theme. In such cases, the tweet was included in multiple themes rather than trying to fit it into one. As a final step, a second sub-sample that includes %15 (*n* = 3259 tweets) of the whole dataset was created by random selection. This sub-sample was also coded by the second author. Interrater reliability analysis was conducted on the second sub-sample to check the consistency of coding. Percent agreement and Cohen’s Kappa values were used as the reliability indices (Gisev et al., 2013). In the Results section, we provide some sample tweets for the detected themes. The tweets were originally in Turkish and translated to English by the authors who are both native Turkish speakers.

## Results

Thematic analysis revealed fifteen distinct themes: These themes were *(1) Support for moving to distance education, (2) Poor institutional infrastructure, (3) Digital inequality, (4) Negative attitudes towards distance education, (5) Pedagogical issues, (6) Concerns about Applied Courses, (7) Assessment Issues, (8) Offering alternatives, (9) Discarding students in decision-making*; (10) *Protesting the decision makers;* 11) *Physical and mental health issues; 12) Financial issues; 13) Social consequences; 14) Emotional expressions, and 15) Humor.* Theme definitions are displayed in Table 1. The interrater reliability scores were high or moderate-to-high across the themes according to the Cohen’s Kappa values (see Table 1).

**Table 1:** Themes, definitions, interrater reliability scores, and frequencies

Category	Description	Percent agreement	Cohen’s Kappa	Frequency
Support for moving to distance education	Tweets support distance education either (1) because there is no other/better option than distance education during Covid-19 crisis; (2) to show support for the Turkish government about the decision.	97.1	0.87	13%
Poor institutional infrastructure	Tweets tell that universities have no technical capability/infrastructure to provide distance education	97.9	0.821	6%
Digital inequality	Tweets underline specific inequalities in the society in terms of accessing distance education (e.g. having computers, internet connection).	96.8	0.874	15%
Negative attitude towards distance education	Tweets state that distance education is inferior to face-to-face education, inefficient, and have no benefit for future career. No justification is provided for the negative attitude.	94.9	0.811	16%
Pedagogical issues	Tweets criticize distance education from pedagogical aspects including low interactivity between teachers and students, poor quality instructional materials, and problems with course planning and management.	97.4	0.679	4%
Concerns about applied courses	Tweets talk about inapplicability of distance education to applied courses	98.7	0.689	2%
Assessment issues	Tweets show worries about fairness of exams/assessment and objective grading	98.7	0,791	3%
Offering alternatives	Tweets suggest specific alternative(s) to distance education	95.3	0.857	16%
Discarding students in decision-making	Tweets claim that students’ views have been ignored when switching to distance education and decision makers should pay attention to the concerns of students	96.9	0.76	7%
Protesting the decision makers	Tweets criticize government and/or other decision makers (e.g. Higher Education Council) about their inability in handling the situation.	99.8	0.92	1%
Physical and mental health issues	Tweets say that current physical and mental health issues in the society hinders distance education	98.4	0.754	3%
Financial issues	Tweets mention financial concerns (e.g. dormitory fees, course fees, and rents) about halting face-to-face education.¨	99.3	0.806	2%
Social consequences	Tweets express dissatisfaction about social/school life consequences of distance education	98.5	0.556	1%
Emotional expressions	Tweets include explicit expression of emotions about distance education (e.g. sadness, anger, crying)	96.5	0.648	6%
Humor	Tweets that include humour/jokes/memes related to distance education	96.6	0.725	5%

*Support for moving to distance education* theme constitutes %13 (n = 2685) of tweets posted under all themes. Twitter users supporting distance education during the Covid-19 pandemic have mentioned several health-related dangers about continuing face-to-face education. The mentioned dangers were generally about high possibility to get infected during face-to-face education, spreading disease to other family members, and dying.

Go to school [i.e. university], and die then.

Why, do you want to get sick?

It was observed that higher education students further expressed support to distance education because they did not want their courses to be postponed to a future date or their graduation to be delayed.

…What do you mean #wedontwantdistanceeducation? Are you nuts? What do you want? Attend courses the whole summer? I don’t understand you… I wont…

The ones who say #wedontwantdistanceeducation, what are your suggestions during this pandemic then? For example, would it fit you if this terms is cancelled, you take all the courses [for this term] next year, and seniors students’ education is extended one more year?

I will graduate [this year]. I would take that course even if offered in Wuhan. Continue with #distanceeducation.

A significant amount of posts under *Support for moving to distance education* theme regarded #wedontwantdistanceeducation hashtag as a criticism to the government. Thus, some tweets displayed support to the government officials about their decision in switching to distance education.

Then, don’t accept the [government] stipends/loans given during this period. No one is doing this [i.e. switching to online education] for pleasure. Do not act as if it is only the students who suffer. This is the first time we experience such a situation. It can’t be foreseen.

F*** off then! The government is always regarded with disfavor, whatever it does.

Opening this tag is pertness, when all the government agencies are now mobilized [to deal with the Covid-19].

*Poor institutional infrastructure* theme formed % 6 (n = 1319) of tweets. Tweets in this theme have revealed that higher education students had severe problems in accessing online courses. This was mainly because universities did not have sufficient technical infrastructure to provide online courses for big masses of students.

No reliable access has been provided to the distance courses. We still can’t access to the course that started on Monday.

For the first time, I follow a course like watching silent motion pictures. There is no voice coming but the slides are moving [on the screen]. It’s like a joke!

The lesson ends before we manage to log in to the system. The screen is always frozen. Please fix the system!

*Digital inequality* theme consisted of % 15 (n = 3084) of the tweets. It has been observed that some higher education students did not have basic necessities (e.g. computers, and internet) to access online education. Thus, online education has been criticized for leaving disadvantaged students out.

What will happen to those who don’t have internet at home? No one cares about them…

My friend does not have a computer, forget about the internet.

… There are even villages with no cell phone connection in this country. Please first provide equal conditions, or stop giving education like this.

*Negative attitudes towards distance education* theme comprises (%16, n = 3331) of all the tweets. Higher education students have stated that distance education is inferior to face-to-face education in terms of overall effectiveness.

Because it is inefficacious.

It can’t be a substitute for face-to-face education.

What? No to superficial education.

You can’t throw a whole education year to the garbage like this. This system you apply [i.e. distance education] will not give any benefit to the students.

It seems that the higher education students’ *negative attitudes towards distance education* is based on the history of distance education in Turkey. In the country, Open Education faculties at several universities have been offering distance education for decades. The tweets show that higher education students regard online education during Covid-19 crisis as a mere replication of distance education offered in Open Education faculties that ask for minimal or no admission requirements.

If we wanted to study from distance, we would have gone to the Open Education [Faculty].

I don’t accept the system that obliges me to study in Open Education although I won formal education.

If I wanted to have a degree by watching videos, I would have studied Open Education.

*Pedagogical issues* constituted % 4 (n = 899) of the tweets. The theme revealed that higher education students were not satisfied with the pedagogy applied in online classes during Covid-19 crisis. For example, a common pedagogical issue reported in the tweets was the low interactivity between teachers, students and the content.

There is no teacher answering our questions. What kind of education is this?

The teacher who gives non-stop lectures for 2,5 hours [in face-to-face education] ended the lesson in half-hour [in online education]

We don’t want to have [online] lessons that is all about uploading slides to the system

So, I paid 9000 Turkish Lira to read pdfs at home?

Teachers send pdfs and show no effort at all!

Another *pedagogical issue* observed in the tweets was the planning and management of online courses. It seems that higher education students were not happy with how some teachers organized their online classes.

So, online education about giving lots of homework and pinning down students?

What the hell is giving an assignment in the morning and asking it to be returned by the evening? I will eat the assignment because of anger.

… when we open [our] microphone [during online classes] they [teachers] try to scold us like kids.

I think teachers misunderstood the distance education. The aim is to give education not punishment.

*Concerns about applied courses* theme consisted of % 2 (n = 373) of the tweets. Higher education students have posted that distance education was not suitable for applied courses.

You can’t teach Medicine with distance education.

In order to attend the Piano classes we have to have a piano…

I study gastronomy… I don’t understand how applied courses will be taught.

*Assessment issues* theme included % 3 (n = 677) of tweets. Higher education students have expressed a variety of concerns regarding the possibility of cheating and unfair grading in online education.

I`m a freshmen in the faculty. Everyone will cheat in the exam. Please don’t allow this.

All those problems that will happen during the [online] exams will impact my grade point averag. How this is gonna be compensated? How will many things like noise in the house, electric black out will affect my grades?

… Grading will be a fiasco. Everyone will pass with AAs. No one can know who is successful who is not.

…Online exams scare us. There will be lots of injustice and we don’t want this to happen.

*Offering alternatives* theme represented % 16 (n = 3352) of the tweets. In this theme, higher education students suggested decision makers to organize summer courses or delay the courses until the pandemic is over.

We even accept delaying of courses to summer. At least we won’t be subject to this [online education] system that has no benefit at all.

Isn’t it better to come to the school in the summer? I can’t follow this [online] education.

Courses should be taught face-to-face when the pandemic is over.

*Discarding students in decision-making* theme included %7 (n = 1401) of tweets. Tweets in this theme has shown that decision makers switched to online learning in a top-down manner without considering students views, expectations and conditions. In addition, many higher education students complained about the chronology of decisions that lead to switching to online education. Higher Education Council first announced a three-week holiday and underlined that there wouldn’t be any teaching during this period. After this announcement, most of the higher education students have returned to their homes leaving their course materials behind. While students were on holiday, Higher Education Council switched to online education. For many students, it was not possible to go back to their universities and pick up course materials. Thus, students ended up having no books, no notes and even no computers to participate in online education.

…If you really want to make up for face-to-face education, pay attention to what students say.

Why can’t universities make their own decisions in collaboration with their student councils? Have you ever asked formal education [i.e. face-to-face] students, even once, about what they want? Or to the academic staff?...

None of our thoughts about education is considered. We are the students, the decision directly effects us, but no one pays regard to students. We send e-mails, write petitions but we can’t reach. The issue ends up in Twitter...

It was announced that there will not be distance education. People returned to their homes at a moment’s notice. Everyone left their notes, books at the dormitories.

*Protesting the decision makers* theme was observed in %1 (n = 251) of the tweets. Under this theme, higher education students raised their voices against the government and the other decision makers such as Higher Education Council.

Now I will sit and watch how much you will make a hash of this [i.e. online education] while sometimes feeling shame and sometimes pleasure.

Wrong decision. You messed up the education.

You are too distant to education…

You can console yourself with the thought that you offered distance education

*Physical and mental health issues* theme comprised %3 (n = 687) of the tweets. It was observed that higher education students have found it challenging to focus on their education under mental and physical health stressors caused by the Covid-19 pandemic.

No one can adapt to distance education with this psychology. Every one is concerned about their health…

Don’t expect us to study while getting death news every day.

…Psychologically, no one is feeling good. While everyone is thinking about their families, how are they gonna successfully learn, attend exams?

…How can you keep teaching at this time? You don’t consider students’ psychology.

Our balance is upside down. We lost our bearings.

*Financial Issues* theme formed %2 (n = 381) of tweets. Higher education students have reported that they have kept paying school fees, rents and other costs about on-campus education although they do not receive it anymore.

Give us back our tuition fees.

Who will pay for the contracts we signed for the flats, dormitories and studios? Are we gonna pay for houses we don’t stay? We left all our books at the dormitories. Are we gonna buy them again? You can’t study without resources. Find a solution to this.

We are paying to private dormitories for nothing.

If I can’t get a face-to-face education in this city, why am I paying rent, bills, dues, and heating fees?

*Social consequences* theme was consisted of % 1 (n = 207) of the tweets. Students have mentioned that online education has ceased their social life at campuses and their graduation ceremony dreams have come to naught.

Man, our [time in school] canteen has oozed away.

Maybe there are some people we would like to see at school.

We would like to say goodbye to our university with a cap and academic gown.

So, I won’t be enjoying my school in my senior year and throw a cap?

*Emotional expressions* represented %6 (n = 1243) of the tweets. The theme showed that higher education students displayed a variety of negative emotions with regards to online education during the Covid-19 pandemic. These emotions included anger, frustration, longing, and sadness.

Missing schools?... Seriously, this made me upset…

We are depleted.

We don’t want we don’t want we don’t want!!

I will break the keyboard on my head because of anger. What the hell is this?

I swear I will cry.

The final theme, *Humour*, represented %5 (n = 1133) of tweets. It was observed that students were expressing their thoughts, feelings and experiences regarding online education through sarcastic posts that sometimes included funny photos and memes.

It’s a shock to the lovers who study in same university but live in different cities. There is no spring term, but only distance education [face with tears of joy emojis].

From now on, we will look for free Internet under the Coca-Cola caps to attend [online] classes.

2019–2020 graduates. [A montaged photo of classmates in hazmat suits with a graduation cap on their head]

## Discussion

The purpose of this study was to explore Turkish higher education students’ views about online education during the Covid-19 pandemic. For this aim, we explored tweets posted under #wedontwantdistanceeducation hashtag that became a popular topic in Twitter after all the Turkish Universities switched to online education with a top-down decision from the government. Based on thematic analysis of tweets, our study revealed that Turkish higher education students did not unanimously approve online education. The students who support online education stated that there is no better option than online education since Covid-19 virus can easily spread during face-to-face education. Students further stated that the end of the Covid-19 pandemic is unforeseen. Thus, delaying of courses might not be a sustainable solution and have consequences such as postponing of graduation date.

Our findings show that Turkish higher education students have an overall negative attitude towards online education. Historically, distance education programs in Turkey had very low admission requirements (Latchem et al., 2009), and many people see distance education as a low quality alternative to on-campus education (Cekerol, 2012). Based on this general view in the society, Turkish higher education students were concerned that distance education would not help with their learning or serve to their career development. It is worthy of noting that Turkish Higher Education Council framed online education during the Covid-19 pandemic as distance education. This framing might have also triggered negative conceptions about online education in time of Covid-19. Nevertheless, the current finding indicate that online education still faces a negative attitude barrier among the higher education students despite its fast diffusion in higher education contexts. Thus, universities should put more effort in changing conceptions about online education in the society in order to attract more online learners.

It is important to develop a solid technical infrastructure for online education (Zheng et al., 2018). Our study has shown the students considered that Turkish universities were caught unprepared to the Covid-19 pandemic in terms of technical infrastructure. In Twitter, higher education students complained about a variety of technical problems with online learning such as crashing websites, log in problems, disconnection during online assessments, and missing audio. These complaints suggest that higher education institutions should invest more in their technical facilities to provide smooth learning experiences and increase learner satisfaction.

The rapid advancement of Covid-19 across the world has pushed people into forced self-isolation. Consequently, digital tools have become a crucial means for the isolated individuals to access to the outer world. However, there are big gaps between and within the societies in terms of digital access to information and resources (Beaunoyer & Dup, 2020). Such inequalities can have serious consequences on people’s social well-being (Büchi et al., 2018). Extending this, the current study have found that the Covid-19 pandemic escalates inequalities in accessing education. Many higher education students did not have internet connection or computers to access education offered by the universities. As a mitigation strategy, Turkish Higher Education Council offered 6 gigabytes free internet connection to all higher education students to access some specific online education platforms (Anadolu Ajansi, 2020d). However, further efforts are necessary to support online learning of disadvantaged students in Turkey and across the world.

Higher education students have reported several pedagogical issues that undermine their online learning experiences during Covid-19 crisis. These issues specifically include low interaction between instructors and students, poor quality course materials and problems in course planning and management. Based on these identified issues, it can be claimed that Covid-19 crisis have caught teachers off guard too. This can be expected since teachers faced a big workload demand to transform their teaching to fully online form. Online and face-to-face teaching are not identical. Online education requires a more thorough planning and a higher time investment in course design and management compared with face-to-face education (Baran et al., 2013). Further, teachers should develop new skills in creating meaningful relationships with students, monitoring student progress, and providing timely feedback in online learning mediums (Philipsen et al., 2019). Skills do not develop over the night. It is unrealistic to expect high quality online education from teachers without providing them training opportunities and support in competence development for online education. During the Covid-19 pandemic, Turkish university teachers were asked to start online teaching in a week after the suspension of face-to-face education. This was like throwing someone who does not know swimming into water and expect him/her to learn swimming by him/herself. Our findings revealed that teachers could not “swim” well, according to their students. Future studies should explore university teachers’ views about the challenges they have faced due to abrupt switching to online education.

Higher education students were explicitly worried about assessment issues in online education. Cheating is a common phenomenon in Turkish universities (Yazici et al., 2011). Thus, students were concerned that it was not possible to detect cheating in online exams. Further, students displayed distrust against their universities’ technical capacity in conducting online exams. Lack of security in online exams is a universal challenge in online education (Xiong & Suen, 2018). Some universities use software that can track students’ facial expressions, voice, location and browsing behaviors in computers to avoid cheating. However, this raises serious issues in terms of students’ privacy. Therefore, it is common to evaluate student performance through individual assignments, question and answer sessions, and peer discussions in online education (Xiong & Suen, 2018). However, fast-track or automated grading cannot be applied to these assessment methods. Therefore, offering reliable and fair assessment to the learners without burdening the teachers remains a big challenge in online education (Akimov & Malin, 2020).

The current findings revealed that switching to online education during the Covid-19 pandemic has hit students from several aspects other than education quality. For example, many students expressed worries about continuing financial burden of suspended face-to-face education. A considerable amount of students have also expressed their disappointments regarding the social consequences of the Covid-19 pandemic such as missing friends and cancelled graduation ceremonies. In addition, students were not able to focus on their education due to mental and physical health issues in themselves or their family members. Students’ worries and disappointments are also reflected as negative emotional expressions in their Twitter posts. Considering this, we suggest that universities should support mental well-being of their students in addition to sustaining education at these exceptional times.

Social media has been a popular civic and political engagement platform for higher education students (Cabrera et al., 2017; Mwangi et al., 2018). Supporting previous work, we have found that Turkish higher education students have been eager in reaching out government agencies (e.g. Turkish Higher Education Council and university administrations) about their expectations and concerns about the online education during the Covid-19 pandemic. Students made it explicit that they are ignored during the decision-making process and suggested some solutions to the ongoing problems with online education (e.g. applied courses). However, students were not united in terms of their demands from government agencies. For example, some suggested courses to be organized in the summer rather than having them online. On the contrary, some argued that organizing summer courses would be a terrible idea. Nevertheless, our findings show that listening to students’ voices in social media can provide valuable insights to government agencies about the impact of their decisions among the students and help to modify their decisions with regards to the multiple perspectives presented by the students in social media.

Finally, the current findings indicate that higher education students’ use of social media is not limited to political or civic engagement. For example, entertainment is a common gratification sought from social media (Author). In the current study, a humor theme was evident in postings under the #wedontwantdistanceeducation hashtag. In these posts, students were making fun of their online learning experiences (e.g. posting gif pictures or memes about how they follow online courses, joking about girl/boyfriends living in different cities, and mentioning some funny nicknames seen in online classes). Thus, it can be concluded that Turkish higher education students use social media for entertainment purposes as well.

The current study reflects Turkish higher education students’ initial responses to the online remote education at the beginning of the Covid-19 pandemic. Since then, a plethora of studies have explored how the sudden switch to online remote teaching has impacted higher education community from various perspectives. Overall, the findings have showed that the remote online education challenges found in this study has had negative impact on students’ well-being (Holm-Hadulla et al., 2021; Lukacs, 2021). High stress, increased burn out and decreased organizational commitment were also observed among the teachers (Akartuna & Serin, 2022; Esici et al., 2021; Pressley et al., 2021). Drawing on this, research community and policy makers have come up with several remedies to tackle the challenges of remote online learning. These remedies included facilitating faster uptake of open educational resources (Stracke et al., 2022), emphasis on professional development of teachers for online education (İbrahim et al., 2022; Rowland et al., 2022), utilizing social media for just-in-time information seeking and knowledge sharing (Carpenter et al., 2021; Dindar & Yaman, 2018; Greenhow et al., 2021), sharing experiences about new ways of online teaching (Biasutti et al., 2022; Infante-moro et al., 2022; Nerantzi, 2020; Sadeck, 2022), and facilitating community building and inclusion in online platforms (Benson et al., 2021). We hope that the challenges faced and the lessons leant during the pandemic time will facilitate development of highly resilient education systems that will support human development and well-being in both physical and online settings.

## Conclusion

Our study shows that the Covid-19 pandemic has been a benchmark to assess Turkish higher education students’ perceptions and experiences about online education, and Turkish universities’ readiness for offering it. By using thematic analysis on Twitter posts under the #wedontwantdistanceeducation hashtag, we have found out that higher education students have a general negative attitude towards online education, and rather prefer face-to-face education. Based on this, we suggest that universities offering online programs or degrees should have a thorough look at the historical and societal reasons underlying those negative attitudes. The current study further identified several challenges that undermine the quality of online learning in Turkish universities during Covid-19 crisis. Based on the challenges reported in this paper, we suggest that universities should develop their technical infrastructure for online education, support university teachers in developing their online teaching skills, and provide secure and fair assessment in online courses. Further, universities should provide support to their students in coping with the mental and physical stress caused by the Covid-19 virus. Our findings further revealed that digital inequality in accessing online education during Covid-19 has had negative impacts on learning of disadvantaged higher education students. Thus, governments should take actions to provide equal access to education among different socio-economical layers of the society.

## Data Availability

The dataset used during the current study is available from the corresponding author upon request.
